# Feasibility of Implementing a Family-Based Inpatient Program for Adolescents With Anorexia Nervosa: A Retrospective Cohort Study

**DOI:** 10.3389/fpsyt.2019.00887

**Published:** 2019-12-03

**Authors:** Wendy Spettigue, Mark L. Norris, Ingrid Douziech, Katherine Henderson, Annick Buchholz, Darcie D. Valois, Nicole G. Hammond, Nicole Obeid

**Affiliations:** ^1^Department of Psychiatry, University of Ottawa, Ottawa, ON, Canada; ^2^Faculty of Medicine, University of Ottawa, Ottawa, ON, Canada; ^3^Children's Hospital of Eastern Ontario Research Institute, Ottawa, ON, Canada; ^4^Department of Pediatrics, University of Ottawa, Ottawa, ON, Canada; ^5^Anchor Psychological Associates, Ottawa, ON, Canada

**Keywords:** eating disorders, adolescents, Family Based Therapy, inpatient treatment, Maudsley model

## Abstract

**Background:** Manualized Family Based Therapy (FBT) is the treatment of choice for adolescent anorexia nervosa, but it is an outpatient treatment. Very little research has examined whether or how the principles of FBT might be successfully adapted to an inpatient setting, and there is little other evidence in the literature to guide us on how to best treat children and adolescents with eating disorders (EDs) while in hospital. This paper describes and provides treatment outcomes for an intensive inpatient program that was designed for the treatment of adolescents less than 18 years of age with severe anorexia nervosa, based on the principles of FBT. Each patient’s family was provided with FBT adapted for an inpatient setting for the duration of the admission. Parents were encouraged to provide support for all meals in hospital and to plan meal passes out of hospital.

**Methods:** A retrospective cohort study was conducted that examined the outcomes of 153 female patients admitted over a 5-year period. Outcome data focused primarily on weight change as well as psychological indicators of health (i.e., depression, anxiety, ED psychopathology).

**Results:** Paired t-tests with Bonferroni corrections showed significant weight gain associated with a large effect size. In addition, patients showed improvements in scores of mood, anxiety, and ED psychopathology (associated with small to medium effect sizes), though they continued to display high rates of body dissatisfaction and some ongoing suicidality at the time of discharge.

**Conclusion:** This study shows that a specialized inpatient program for adolescents with severe EDs that was created using the principles of FBT results in positive short-term medical and psychological improvements as evidenced by improved weight gain and decreased markers of psychological distress.

## Introduction

Eating disorders (EDs) are serious and potentially life-threatening conditions that typically have their peak age of onset in adolescence (1), with anorexia nervosa (AN) being the third most common chronic illness in adolescent females (2). EDs are associated with significant medical complications including cardiovascular dysfunction, growth retardation, pubertal delay, and low bone mineral density (3). Psychiatric co-morbidities are also common, including depression, anxiety disorders, and substance abuse, and there is a significantly increased risk of completed suicide in this population (4, 5).

Despite their prevalence and seriousness, there is debate about how best to treat these illnesses in young people. Manualized Family Based Therapy (FBT) (6) has the most evidence for weight restoration and long-term recovery in adolescent AN (6–13). This outpatient approach is designed to externalize the illness, lift blame, raise parental anxiety about the seriousness of the illness and the need for weight gain, and empower parents to support recovery at home by taking charge of their child’s nutrition (14). Despite the established efficacy of FBT, as many as 50–65% of adolescent patients with AN may not successfully reach full remission with this approach (15), and the evidence is less clear about the most efficacious form of treatment for these patients. Also, since FBT is an outpatient treatment, little research has examined whether or how the principles of FBT might be successfully adapted to an inpatient setting, and there is little other evidence in the literature to guide us on how to best treat children and adolescents with EDs while in hospital. A recent article by Murray et al. (16) outlined a theoretical framework of how to adapt FBT to higher levels of care, including inpatient admissions. Suggestions included taking the opportunity to raise parental anxiety at the onset of admission, empowering parents to start making choices over their child’s nutrition while in hospital, and using weight gain after weekend passes as indices of parental effectiveness prior to discharge (16). Rhodes and Madden (2) describe the changes they made to their pediatric ED inpatient program to make it more consistent with FBT, but they did not include any outcome data.

These conceptual reports are useful as practical guidelines about how a program might implement FBT in more intensive treatment settings. Our goal in the current study is to provide preliminary data related to the feasibility and outcome of FBT in such a program. As such, this study aims to describe and examine outcome data for 153 female patients admitted over a 5-year period to a specialized pediatric tertiary care inpatient ED program that was designed and adapted around principles of FBT. Patients who underwent a formal assessment by our team and were admitted to the ED inpatient unit were compared on weight and ED psychopathology outcomes from pre– to post–inpatient admission.

## Materials and Methods

### Inpatient Program

Our specialized ED inpatient program is located in a tertiary children’s hospital and contains six dedicated beds on a psychiatric unit for children and adolescents up to age 18 years with severe EDs. The program is staffed by a multidisciplinary team including psychiatrists, psychologists, adolescent health physicians, and dieticians, among other professionals with specialized training in EDs. Most patients live in the surrounding area or within a 150 km radius of the hospital, covering a catchment area of almost 1 million people. Generally, these adolescents are voluntary but are brought to hospital by their parents and are not motivated for treatment; they are admitted to the intensive inpatient program because they are medically unstable (low weight and/or with unstable vital signs, usually bradycardia), are highly symptomatic, and/or have failed outpatient treatment, and do not meet criteria for less intensive levels of the program’s services such as day hospitalization.

The overall aim of our “FBT-informed” inpatient program is to empower parents to compassionately take charge of their child’s nutrition (6, 17). This task is accomplished *via* the use of FBT-based treatment goals of lifting guilt and raising anxiety about the seriousness of the child’s illness, in addition to educating parents regarding EDs and helping them to provide meal support to their child, first in hospital and then out of hospital on passes. The program empowers parents by asking them to be present as much as possible throughout the admission, starting FBT with an assigned therapist, including parents in nutritional planning sessions, and providing education around EDs and meal support using expert-endorsed books, videos, and, at times, a parent support group. Each family receives a family therapist (either a psychiatrist or a psychologist) specialized in the treatment of EDs. The therapist meets at least once a week with the family, and sometimes more often. The therapist may choose to see the parents alone, or with the child, or both. Family therapy sessions focus on lifting guilt and blame, raising anxiety about the dangers of starvation and the importance of renourishment, externalizing the illness, and empowering parents to “keep their child safe” from the ED, to “stand up to the bully that has taken over their daughter’s brain,” and to prevent the illness from “destroying” their child’s mental and physical health and well-being. Passes begin as soon as medical stability occurs so that the patient can spend time with family at home, first between meals, then gradually for longer periods of time that include meals. Parents are encouraged to plan the passes, and attending physicians are flexible and supportive in encouraging decisions around such pass-planning. At home, parents practice food selection, preparation, and serving of meals while encouraging their children to take the necessary nutrition and compassionately containing symptoms (for example, if a patient has symptoms of exercising at night, the parents and siblings will work together in a family therapy session to decide how to keep the patient “safe” from these symptoms when on pass, including possibly choosing which family member will sleep with the patient). Patients work on nutrition goals using in-hospital experiences and out-of-hospital passes and are discharged after completion of at least one successful extended pass home (e.g., taking all nutrition over 2–3 days).

Most parents respond extremely well to FBT during the admission, and do an excellent job of supporting their child compassionately, taking control of nutrition, supervising meals in hospital, and planning passes. Some express fear of discharge, due to a fear that the ED will worsen after discharge and the patient will relapse, so therapists have to work hard to empower parents and help them to feel stronger than the ED and not dependent on the hospital. Admittedly, some parents use the “threat” of returning to hospital to try to get their child to eat. One area of contention is meal plans. Most patients are medically unwell and are put on non-selective menus at admission, followed eventually by selective menus with parents choosing the nutrition, plus choosing the nutrition on passes. However, many parents and patients feel “comforted” by the meal plan and choose to follow it after discharge, which can lead to a reliance on the hospital dietician for increases in nutrition, rather than parents feeling empowered to make the necessary changes. Other parents are able to use the meal plan as a guideline for in-hospital only, and are able to take charge of the type and amount of nutrition after discharge. Another challenge is when parents are unable to come to the hospital to support their child. This is especially problematic for single parents who work, or who don’t drive a car and live far from the hospital. For example, a single mother who runs a home day care will have great difficulty getting to the hospital to supervise meals or attend family therapy sessions. Their children often end up eating meals with staff, and can be acutely aware that other patients are spending time with their families. Through no fault of the parent, the inpatient program is less helpful for these patients, who can end up enjoying the groups, bonding with the staff, doing poorly on passes home, and resisting discharge. Therapists must work hard to come up with innovative solutions for these families, including calling on help from their relatives and friends if possible, and using emails and phone calls to communicate with parents.

### Patient Population

We included data from the charts of females with AN sequentially admitted to our inpatient EDs unit between April 2008 and October 2013. Outcomes from 153 patients between the ages of 13 and 18 years were retrospectively reviewed.

#### Diagnostic Categories

Patients were diagnosed (based on consensus diagnosis by an adolescent health physician and a psychiatrist or psychologist) using information gained from a clinical interview of the patient and the parents (interviewed separately), psychological measures [including the Eating Disorder Examination Questionnaire—Adolescent Version (EDEQ-A), Eating Disorder Inventory (EDI), Children’s Depression Inventory (CDI), and Multidimensional Anxiety Scale for Children (MASC)], and medical history and data. Patients were then classified into one of two diagnostic categories: those with restrictive EDs, all of whom would now meet *DSM-5* criteria for AN—Restrictive subtype (AN-R); and low-weight patients with binge–purge behaviors, all of whom would now meet *DSM-5* criteria for Anorexia Nervosa—Binge/Purge subtype (AN-BP).The study was approved by the hospital’s Research Ethics Board.

### Measures

Well-validated self-report questionnaires were administered by a trained psychometrist. Demographic characteristics were collected using a self-report parent intake form, developed by members of the ED program. For the purposes of this study, parental employment, primary language spoken at home, and whether the child/adolescent has an identified learning disability or individualized education plan (IEP) were examined.

#### Weight Gain

A patient’s height is taken at admission, and weight is taken daily, including at admission and discharge, by a nurse using a calibrated scale (patient is in a gown, post voiding, with back to the scale). Body mass index (BMI) is used as a marker of medical status and used to track weight gain and proximity to the patient’s treatment goal weight (TGW) range. Using the publicly available World Health Organization’s (18, 19) Child Health Growth References macros for the IBM Statistical Package for the Social Sciences (SPSS), BMI-for-age z-scores were calculated. Cutoff interpretations are as follows: normal ≤ +1 standard deviation (SD) to ≥ −2 SD; thinness < −2 SD; severe thinness < −3 SD (20).

#### Eating Behaviors and Attitudes

The EDEQ-A was used to measure ED psychopathology. As the EDEQ-A was only administered to patients 13 years of age or older due to developmental appropriateness, children <13 years were excluded from analyses. The EDEQ-A is a self-report questionnaire with 36 items, that produces a global score (average of the four subscales) and four subscale scores (Restraint, Eating Concern, Shape Concern, and Weight Concern) and has been found to have strong psychometric properties (21).

The Eating Disorder Inventory-3 (EDI-3) (22) was administered to measure ED attitudes and cognitions. The EDI-3 discriminates between samples (clinical versus nonclinical) and diagnoses [AN, bulimia nervosa (BN), partial AN/BN], and is documented to have good internal reliability for ED patients (α = 0.76–0.92) (23). The drive for thinness and body dissatisfaction subscales were used in this study.

#### Psychological Functioning

Depressive symptoms were measured using the CDI (24) and levels of anxiety with the MASC (22). The CDI total score is reported to have excellent internal consistency (Cronbach’s α) in a clinical sample of females diagnosed with an ED (α = .93) (23). The MASC total score has high internal reliability in community samples (α = .90) and excellent internal consistency within clinical samples of children and youth with EDs (α = .92) (25). For the purposes of this study, only the total scores from the measures of depression and anxiety were used in our analyses. In addition, suicidality was assessed using a single item on the CDI (question #9) that asks respondents to indicate their level of suicidal ideation over the past 2 weeks.

## Statistical Analyses

Analyses were conducted using IBM SPSS Version 22. To examine psychological and weight change from admission to discharge, we performed Bonferroni-corrected paired-samples t-tests. Cohen’s d (standardized mean difference) was the measure of effect size computed using Comprehensive Meta-Analysis (Version 3). As suggested by Cohen (26), d = .20 represents a small effect, d = .50 represents a moderate effect, and d = .80 represents a large effect.

Missing data analyses revealed that 67.7% (*n* = 109) of the original sample had self-report pre- and post- data available. The demographic and clinical characteristics of patients with missing data on all psychological measures at either time point (*n* = 52, 32.3%) were compared to patients included in the analysis of one or more psychological outcomes (*n* = 109, 67.7%). Bonferroni-corrected comparisons showed that included versus excluded patients did not differ on age or BMI at time of admission (*p* = .104 and *p* = .049, respectively), nor did they differ on some of the demographic characteristics listed in **Table 1** (range of *p* values:.071 to 1.00). Of note, patients with missing data on all psychological outcomes had a significantly shorter length of stay (LOS) (*M* = 33.06, *SD* = 26.06) than those who completed measures at pre- and post- on one or more self-report measures (*M* = 55.16, *SD* = 20.48, *p* < .001). Patients with missing data were dropped from analyses that included variables that they were missing.

**Table 1:** Parent (*n* = 133) Self-Report Demographics and Family Demographics at Inpatient Admission.

Demographic Characteristics	*n*	%
Primary language:		
English	112	69.6
French	11	6.8
Married parents	77	57.8
Working parents	90	55.9
Individualized education plan	19	11.8
Learning disability	12	7.5
Family history of:		
AN	24	14.9
BN	15	9.3
BED	7	4.3
Depression	89	55.3
Suicide attempts	33	20.5
Severe anxiety	50	31.1

Primary language = primary language spoken at home; working parents = both parents employed full- or part-time; AN, anorexia nervosa; BN, bulimia nervosa; BED, binge-eating disorder.

## Results

### Demographics and Clinical Characteristics

Demographics and clinical characteristics at admission can be found in **Table 1**.

#### Family Demographics

Parent self-report demographics are reported in **Table 1**. English was the most common primary language spoken at home (69.6%), and interestingly, only a minority of patients lived with married parents (47.8%). Most families reported having two working parents, full-time or part-time (55.9%). At assessment, 11.8% of parents reported their child as having an IEP and 7.5% of patients as having a recognized learning disability. Family lifetime prevalence of mental health disorders was collected *via* parent self-report at the time of admission. Family lifetime prevalence of depression was 55.3%, and lifetime family prevalence of any ED was 24.8%, with the most common self-reported ED within a family being AN (14.9%).

#### Demographics of the Diagnostic Categories

Patient clinical characteristics of the 153 females included in the study are presented in **Table 2**. Of the sample, 76.5% (*n* = 117) met criteria for *DSM-5* AN-R and 23.5% (*n* = 36) met criteria for AN-BP. Average age for the sample was 15.26 years of age (SD = 1.74), and mean LOS in hospital was 48.90 days (SD = 24.60). The average LOS was 48.22 days (SD = 24.37) for AN-Rs and 51.11 days (SD = 25.54) for AN-BPs. The AN-BPs were the oldest group (15.75 years of age) with the longest LOS (51.11 days).

**Table 2:** Paired-Samples t-Test Results for Inpatient Admission to Discharge by Subgroup.

Subsample	Outcome	Admission		Discharge		*n*	95% CI	*t*	*p*	*d*
		*M*	*SD*	*M*	*SD*					
AN-R										
	Age	15.11	1.84			117				
	BMI	16.49	2.19	19.0	2.08	117				
	zBMI	−1.70	1.15	−0.51	0.91	117	−1.33, −1.06	−17.43	.000*	1.11
	EDI: drive for thinness	19.0	9.25	16.02	9.88	43	0.39, 5.57	2.32	.025	0.31
	EDI: body dissatisfaction	24.07	12.57	26.40	13.17	43	−5.00, 0.35	−1.76	.086	−0.18
	CDI: total T score	63.79	18.09	55.52	17.72	73	4.78, 11.77	4.72	.000*	0.46
	MASC: total T score	59.87	10.15	54.62	12.65	71	3.17, 7.33	5.04	.000*	0.44
	EDEQ-A: total score	3.18	1.85	2.26	1.85	66	0.55, 1.30	4.90	.000*	0.50
AN-BP										
	Age	15.75	1.27			36				
	BMI	17.80	2.39	20.23	2.09	36				
	zBMI	−1.21	1.02	−0.18	0.72	36	−1.27, −0.80	−8.89	.000*	1.08
	EDI: drive for thinness	20.56	9.62	11.33	9.63	9	2.21, 16.23	3.03	.016	0.96
	EDI: body dissatisfaction	27.67	14.77	23.11	14.10	9	−1.06, 10.17	1.87	.098	0.32
	CDI: total T score	77.72	15.31	69.16	19.30	25	1.45, 15.68	2.48	.020	0.48
	MASC: total T score	62.04	10.69	57.04	14.89	24	0.67, 9.37	2.37	.027	0.36
	EDEQ-A: total score	4.35	1.57	3.37	1.99	22	0.27, 1.69	2.87	.009	0.54

AN-R, Anorexia Nervosa—Restrictive; AN-BP, Anorexia Nervosa—Binge/Purge; BMI, body mass index in kg/m2; zBMI, body mass index for age z-scores; EDI, Eating Disorder Inventory; CDI, Children’s Depression Inventory; MASC, Multidimensional Anxiety Scale for Children; EDEQ-A, Eating Disorder Examination Questionnaire—Adolescent Version; CI, confidence interval. * Significant at p < .003 (Bonferroni corrected).

### Changes From Admission to Discharge

#### Weight Gain

Our findings show that both AN-R and AN-BP patients experienced significant increases in BMI for age z-scores (zBMI) from admission to discharge (*p* < .001; see **Table 2**). Patients with AN-R gained an average of 6.54 kg over 48.22 days (mean 0.95 kg/week), increasing the mean BMI from 16.49 (zBMI −1.70) to 19.0 (zBMI −0.51) at discharge. Patients with AN-BP gained an average of 6.42 kg during admission, increasing the mean BMI from 17.80 (zBMI −1.21) to 20.23 (zBMI −0.18). Weight gain was associated with a large effect size for both ED groups (*ds* = 1.08–1.11). Note that, given the low amount of nutrition prescribed in the initial days of the admission, as per standard protocol at the time of study inclusion, many patients did not start to gain weight until the second week, when the amount of nutrition started to increase beyond 2,000 kilocalories. (As per current recommendations, we now have a more aggressive renourishment protocol.)

#### Eating Behaviors and Attitudes

In terms of ED psychopathology, there was a significant decrease in ED symptoms as measured by the EDEQ-A in the AN-R cohort (*p* < .001; CI: 0.55, 1.30; see **Table 2**). There was a nonsignificant decrease in drive for thinness for the AN-R and the AN-BP groups, with a medium effect size for the AN-R group (*d* = 0.31) and a large effect size for the AN-BP group (*d* = 0.96). Those with AN-BP also had a nonsignificant improvement on the EDI-3 body dissatisfaction subscale (*p* = .098; CI: −1.06, 10.17; see **Table 2**) and a nonsignificant decrease in ED symptoms (*p* = .009; CI: 0.27, 1.69). Improvement in body dissatisfaction was associated with a small effect size (d = 0.32), and reduction in ED symptoms was associated with a medium effect size (*d* = .54). Lastly, there was a nonsignificant increase in body dissatisfaction in those with AN-R (*p* = .086; CI: −5.00, 0.35), with a small effect size (*d* = −0.18).

#### Psychological Functioning

Depression scores significantly decreased (*p* < .001; CI: 4.78, 11.77) from admission (*M* = 63.79, *SD* = 18.09) to discharge (*M* = 55.52, *SD* = 17.72) for those with AN-R (see **Table 2**). Those with AN-R also showed a significant decrease in symptoms of anxiety (*p* < .001; CI: 3.17, 7.33; see **Table 2**). Decreases in symptoms of depression and anxiety were associated with small effect sizes (d = 0.44–0.46). In addition, there was a nonsignificant decrease in depression (*p* = .02; CI: 1.45, 15.68) and anxiety scores for the AN-BP cohort after Bonferroni adjustment (*p* = .027; CI: 0.67, 9.37), both associated with small effect sizes (*d* = 0.36–0.44).

At admission, 38.41% (*n* = 58) of those patients who completed the CDI (*n* = 151) endorsed suicidality (e.g., “I want to kill myself” or “I think about killing myself but would not do it”). Despite the improvement in depression scores, upon discharge, 34.55% (*n* = 38) of 110 patients still endorsed some degree of suicidality.

## Discussion

This study shows that a specialized inpatient program that combines medical treatment, nutritional rehabilitation, psychoeducation, and compassionate containment of symptoms with individual, family, and group therapy to treat adolescents with EDs is effective in achieving improvements in medical stabilization and weight gain, ED symptoms, and psychological functioning. Underweight patients experienced an improvement in medical status and an increase in BMI during the admission. Weight restoration and medical stabilization are of primary importance in the treatment of EDs (27, 28). Patients were able to gain weight adequately within this model of using parents and/or unit staff for meal support.

There is some limited literature looking at the effectiveness of inpatient treatment for adolescent AN. Some of these papers describe cognitive-behavior therapy-informed inpatient treatments, some look at predictors of outcome, and others compare inpatient treatment to day treatment program, or longer lengths of inpatient admission with shorter lengths of stay. Herpertz-Dahlmann et al. (29) compared a longer inpatient treatment for adolescent AN to a shorter inpatient stay (3 weeks) followed by treatment in a specialized EDs day program (29). Eighty-five adolescents were randomized to a longer inpatient stay, and 87 were allocated to day treatment. Results showed no significant differences in BMI at 12-month follow-up, suggesting that a specialized day treatment program for adolescents with AN may be effective and cost-saving compared to inpatient treatment (29). Madden et al. (30) assigned 82 adolescents with AN and medical stability to a short hospitalization, for medical stability (MS), or to a longer admission until they reached 90% of goal weight (WR), with both groups treated on a specialized pediatrics medical ward in one of two hospitals in Australia (30). Both groups were treated with 20 sessions of manualized FBT following discharge. Results at 12-month follow-up showed no difference in outcome between groups, (30% full remission for the MS group, 32.5% full remission for the WR group), but with obvious cost savings for those who had a shorter inpatient stay (30).

Schlegl et al. (31) examined predictors of outcome for 238 adolescents with severe AN treated in an inpatient setting in Prien, Germany (31). Overall, the patients gained weight, with BMI increasing from a mean of 14.8 to 17.3 kg/m^2^; 45% of patients showed significant improvements on the EDI-2. They described their specialized ED inpatient treatment program for youth 14–17 years as one offering specialized individual and group manualized cognitive–behavioral therapy, along with some additional social skills training, art therapy, cooking training, and “sports therapy.” They described parents as “actively involved in the therapy in the form of telephone consultations and family therapy sessions. There are at least three sessions with the nuclear family, one at the beginning of treatment, one in the middle of treatment and one at the end of treatment. Sessions include elements such as family diagnosis and family sculpture, role-playing techniques to clarify family interactions, homework and themes like detachment from family.” They concluded that this program was effective in treating about two-thirds of adolescents with AN (31).

Salbach-Andrae et al. (32) examined outcomes from 57 female adolescents with AN treated at their specialized EDs inpatient unit in Berlin, Germany (32). The 12-week program was described as using a cognitive–behavioral approach including individual therapy, body-image and body-esteem group, expressive art, family therapy (once a week), recreation therapy, and nutritional education. Their results showed that at 1 year, 59.6% of the inpatients showed a poor outcome, and only 28.1% had recovered fully (32).

Hibbs et al. (33) studied an intervention for caregivers whose children were being treated in hospital for an ED, but in various non-specialized ED settings (33). The intervention consisted of providing a book, a DVD, and five coaching sessions to parents whose children were receiving inpatient treatment for an ED. They described “small improvements in symptoms and bed use” in the intervention use and suggested that providing parent support and psychoeducation may be helpful (33).

Halvorsen et al. (34) described an inpatient program in Oslo, Norway, that, like ours, is based on “Maudsley” principles of family-based treatment (34). Unlike our program, where patients may or may not have received previous outpatient treatment, their patients had all been unsuccessfully treated with previous inpatient or outpatient treatment, including family-based interventions. They describe their FBT-inspired inpatient program, in which parents stayed on the unit with the child, as “designed to help parents establish clear, predictable frameworks for meals with adequate amounts of food at the hospital and at home. The family ate all meals together in a designated family room or a designated table in the main dining room, receiving support from the staff as needed. Milieu therapy staff aimed to support the whole family in coping with the ED…. A weekly weight gain of approximately 1 kg was recommended. The family reviewed daily progress with the staff, attended joint family sessions once or twice a week, parental counselling and weekly group sessions for parents. Most patients were also offered weekly individual sessions. Weekend leaves were integrated into treatment to encourage families to practice and transfer skills to the home environment, with longer leaves granted toward the end of hospitalization” (34). The authors contacted 37 adolescents 1–7 years after discharge from their program and reported that only 36% (*n* = 12) were classified as fully recovered (as defined by BMI ≥18.5, EDEQ global ≤2.5, and no binge-eating/purging over past 3 months) (34).

The program described by Halvorsen et al. sounds very similar to ours, other than the fact that in our program, parents sleep at home or at a nearby “Rotel” beside the hospital and eat alone as a family, rather than in a group dining hall. Ours is thus the second study to evaluate an inpatient program for youth with EDs that has integrated FBT principles throughout programming, by having parents support a large proportion of meals while the adolescent is in hospital, having parents work closely with dietitians and plan nutrition on passes, and by offering weekly FBT-informed family therapy sessions with a psychologist or psychiatrist (i.e., FBT modified for an inpatient setting). Unlike the study by Halvorsen et al., which reports long-term outcomes for 37 adolescents, our study does not report long-term outcomes, but describes positive outcomes at discharge for 109 adolescent females (34).

Our results show an improvement in both depressive and anxiety symptoms during the course of the admission. This finding is in keeping with previous literature (35–38) showing that anxiety and depressive scores are highest in malnourished patients and that symptoms improve at least partially with weight restoration. Although weight gain likely contributed to improvements in mood, other factors including the intensive individual, group, and family therapy, in combination with the compassionate and informed therapeutic milieu, would likely have also impacted mood and anxiety scores. Despite significant improvements in mood, one-third of the sample expressed ongoing suicidality at the time of discharge. While we can only speculate, this could possibly be related to the stress of discharge and to ongoing body dissatisfaction. This study used the EDI-3 to assess changes in ED psychological functioning (attitudes and cognitions) pre- and post-admission, and focused on two subscales, drive for thinness and body dissatisfaction.

Our finding that body dissatisfaction did not decrease during admission in those with AN-R is in keeping with previous literature which has shown that physical symptoms tend to remit before psychological symptoms (39). Most patients who are admitted have severe distortions in how they see their body, along with a severe fear of weight gain. They are then required to increase nutrition to bring weight back into a healthy range, usually in a relatively short period of time. Therefore, it is not unusual for a patient’s dissatisfaction with their body shape to increase with weight gain in the early stages of recovery. Surprisingly, drive for thinness showed a downward trend with treatment. It is also important to note that changes in ED cognitions in response to treatment are often delayed in adolescents with AN who are treated with FBT. Thus, given the short duration of treatment examined in the present study (average LOS being approximately 7 weeks), it may not be all that surprising that some observed changes were not significant or were associated with small effect sizes.

### Limitations

One limitation of this study was missing data on some discharge measures (e.g., the EDI). While most pre-admission questionnaire packages are completed at the initial intake assessment successfully, administration of questionnaire packages at discharge was more frequently missed. This can be explained as a result of either patient unwillingness to complete questionnaires, unexpected discharges occurring during off hours (e.g., evening and weekends) when our psychometrist is not available, or sometimes due to busy clinicians neglecting to inform the psychometrist of upcoming discharges. Although missing data analyses revealed no significant differences on demographic and psychological measures at admission between those with and without discharge data, and as this type of missing data is not abnormal in clinical outcomes research settings, some caution is warranted when interpreting the generalizability of the results.

Other limitations include the exclusion of males and diagnoses other than AN (i.e., BN), use of self-report measures and lack of a standardized structured or semi-structured interview to obtain diagnoses, along with limited medical data, no pre- and post- measures of parent satisfaction and empowerment against the ED, lack of long-term follow-up, and the use of a single site which may limit generalizability to other ED programs. An additional limitation is the lack of a comparison group and a non-randomized design. Despite the noted limitations, this study provides initial treatment outcomes on an inpatient program for adolescents that was designed and implemented in the era of burgeoning FBT evidence.

### Future Directions

The current study utilizes data collected between 2008 and 2013. Since that time, our inpatient program has undergone significant changes in an attempt to continue to align more closely with evidence-based FBT principles. As appropriate, lengths of stay are limited and patients are discharged at lower weights to continue treatment using outpatient FBT. Patients spend more time with families and less time in a group milieu, and individual therapy is limited. As well, renourishment protocols are now more “aggressive,” in keeping with updated pediatric guidelines on the nutritional management of inpatient EDs (40). Moving forward, it will be critical to examine how inpatient programs that admit patients who have failed FBT or other traditional outpatient models can achieve success in helping to support a child’s recovery, while taking into consideration indicators such as LOS, weight gain, changes in psychological measures, and most importantly, sustained markers of recovery including long-term outcomes.

## Conclusion

This specialized intensive inpatient program for youth with severe EDs provided psychoeducation and medical and nutritional treatment, in combination with group, individual, and FBT-informed family therapy. Short-term outcomes show that an intensive inpatient program such as this one for youth with severe EDs is successful in restoring weight for patients, and in improving mood, anxiety, and ED symptomatology.

## Data Availability Statement

The datasets generated for this study are available on request to the corresponding author.

## Ethics Statement

This study involving human participants was reviewed and approved by the Research Ethics Board, Children's Hospital of Eastern Ontario Research Institute. Written informed consent to participate in this study was provided by each participant and by their legal guardian/next of kin.

## Author Contributions

WS, KH, and AB proposed, sought funding for, and created the inpatient program described, and provided the individual, group, and family therapy to patients and families. NO created the database, and with WS, KH, and AB, chose the measures for evaluating outcomes of the program. NO collected the data and analyzed it. ID, WS, and MN wrote the manuscript for the paper. NO, NH, and DV helped to analyze the data and describe the results. DV helped with references and edited and formatted the manuscript. All authors have contributed to the writing of this manuscript, and have read and approved of this article’s submission.

## Conflict of Interest

The authors declare that the research was conducted in the absence of any commercial or financial relationships that could be construed as a potential conflict of interest.
